# ZHX2 enhances the cytotoxicity of chemotherapeutic drugs in liver tumor cells by repressing MDR1 via interfering with NF-YA

**DOI:** 10.18632/oncotarget.2832

**Published:** 2014-11-26

**Authors:** Hongxin Ma, Xuetian Yue, Lifen Gao, Xiaohong Liang, Wenjiang Yan, Zhenyu Zhang, Haixia Shan, Hualin Zhang, Brett T. Spear, Chunhong Ma

**Affiliations:** ^1^ Key Laboratory for Experimental Teratology of Ministry of Education and Department of Immunology, Shandong University School of Medicine, Jinan, Shandong, P.R. China; ^2^ Department of Microbiology, Immunology & Molecular Genetics and Markey Cancer Center, University of Kentucky College of Medicine, Lexington, KY, USA

**Keywords:** ZHX2, transcriptional repression, chemoresistance, p-glycoprotein, apoptosis

## Abstract

We previously reported the tumor suppressor function of Zinc-fingers and homeoboxes 2 (ZHX2) in hepatocellular carcinoma (HCC). Other studies indicate the association of increased ZHX2 expression with improved response to high dose chemotherapy in multiple myeloma. Here, we aim to test whether increased ZHX2 levels in HCC cells repress multidrug resistance 1(MDR1) expression resulting in increased sensitivity to chemotherapeutic drugs. We showed evidence that increased ZHX2 levels correlated with reduced MDR1 expression and enhanced the cytotoxicity of CDDP and ADM in different HCC cell lines. Consistently, elevated ZHX2 significantly reduced ADM efflux in HepG2 cells and greatly increased the CDDP-mediated suppression of liver tumor growth *in vivo*. Furthermore, immunohistochemical staining demonstrated the inverse correlation of ZHX2 and MDR1 expression in HCC tissues. Luciferase report assay showed that ZHX2 repressed the MDR1 promoter activity, while knockdown of NF-YA or mutating the NF-Y binding site eliminated this ZHX2-mediated repression of *MDR1* transcription. Co-IP and ChIP assay further suggested that ZHX2 interacted with NF-YA and reduced NF-Y binding to the *MDR1* promoter. Taken together, we clarify that ZHX2 represses NF-Y-mediated activation of *MDR1* transcription and, in doing so, enhances the effects of chemotherapeutics in HCC cells both *in vitro* and *in vivo*.

## INTRODUCTION

Hepatocellular carcinoma (HCC) is one of the most common cancers worldwide with high mortality, and poor prognosis. Although a large number of therapeutic agents have been evaluated for the treatment of HCC, most have been ineffective due to the high chemoresistance, especially the multidrug resistance (MDR) of liver cancer cells [1, 2]. Overexpression of multidrug resistance protein 1(MDR1, also known as p-glycoprotein), an ATP-dependent pump, causes the efflux of various hydrophobic compounds and xenobiotics leading to MDR [3]. Accumulated data confirm the important role of MDR1 in the resistance of HCC cells against different chemodrugs, including the commonly used chemotherapeutic drugs cisplatin [cis-diamminedichloroplatinum (CDDP)] and Adriamycin (ADM)[4-6]. Strategies targeting MDR1 have been recognized as a potential method to restore chemotherapeutic sensitivity of cancer cells, however, strategies have had minimal clinical benefit [3, 7]. Therefore, understanding the regulation of MDR1 expression in HCC is essential to develop more effective treatments.

Previous studies have identified a number of transcription factors binding sites in *MDR1* promoter. A conserved *CCAAT* element (*Y-box*), located between −82 to −73 in the human *MDR1* promoter is absolutely required for basal and inducible expression of the human *MDR1* gene [8, 9]. The nuclear protein NF-Y, a complex consisting of A, B, and C subunits, recognizes the *Y-box* sequences and orchestrates *MDR1* promoter activation [9, 10]. The identification of NF-Y as a central mediator of MDR1 activation makes it an attractive molecular target for manipulating the MDR phenotype and therapeutic intervention.

The *Zinc-Fingers and Homeoboxes 2* (*ZHX2*) gene is a member of a small gene family that also includes *ZHX1* and *ZHX3* [11]. Two-hybrid studies indicate that *ZHX2* can form homodimers as well as heterodimers with other ZHX family members and with NF-YA [12]. Consistent with these data, ZHX2 regulates the NF-YA-dependent genes *cdc25C* and *Hexokinase II* (*HKII*) and has been implicated in cell cycle control [12, 13]. A potential role for ZHX2 in HCC came from studies showing that ZHX2 represses expression of alpha-fetoprotein (AFP), glypican-3 (GPC3) and H19, three genes that are frequently activated in HCC [14, 15]. We recently demonstrated that ZHX2 overexpression leads to G1 arrest and down-regulation of cyclin A and cyclin E expression in HCC cell lines [16]. Altered ZHX2 expression has been reported in hepatic and hematological malignancies [17-19]. Interestingly, high ZHX2 expression is significantly associated with an improved response and longer survival after high dose-chemotherapy in patients with multiple myeloma [17], suggesting that ZHX2 might influence drug resistance in cancer cells.

Based on the function of ZHX2 as a transcriptional repressor and its known interaction with NF-YA, we hypothesized that ZHX2 might inhibit MDR1 expression in liver cancer cells, resulting in reduced efflux of chemotherapeutic drugs and subsequent increased sensitivity to these agents. To test this, the correlation of ZHX2 and MDR1 expression was evaluated in HCC tissues. Then ZHX2 levels were experimentally increased or decreased in several liver cancer cell lines, followed by treatment with CDDP and ADM. Our results indicate that higher ZHX2 levels reduced MDR1 expression and decreased drugs efflux in all HCC lines tested. Consistently, ZHX2 significantly enhanced the sensitivity of HCC cells to chemotherapeutic drugs both *in vitro* and *in vivo*. Taken together, our data identify MDR1 as a new target of the tumor suppressor ZHX2 and suggest that ZHX2 maybe a novel target for the treatment of liver cancer.

## RESULTS

### The expression level of ZHX2 in HCC tissues negatively correlates with that of MDR1

**Figure 1:**
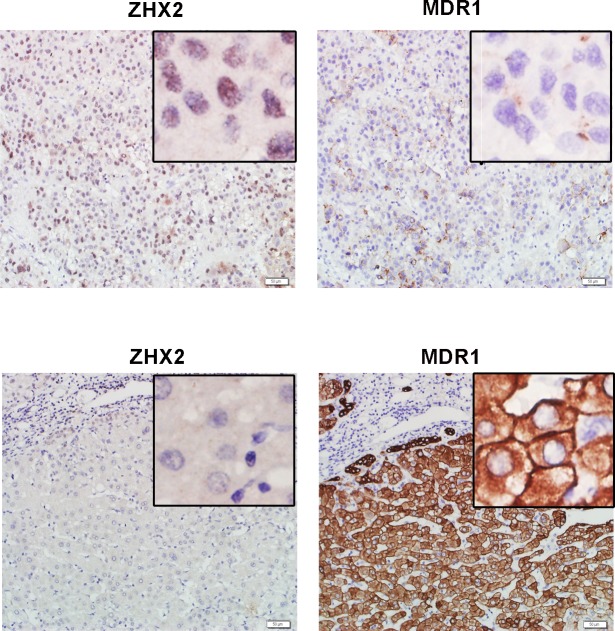
ZHX2 expression is inverse correlated to the expression of MDR1 in HCC Immunohistochemical staining of ZHX2 and MDR1 in adjacent sections of cancer biopsies from 2 of the 30 HCC samples (upper panels, ZHX2 high and MDR1 low ; lower panels, ZHX2 low and MDR1 high). bars = 50μm; statistical results were shown in Table 1.

We first evaluated the correlation of ZHX2 and MDR1 expression in HCC tissues. To address that, thirty HCC samples were involved to do immunohistochemical staining with antibodies against ZHX2 and MDR1. Consistent with our previous study [16], nucleic ZHX2 could be detected in less than 35% (9/30) involved HCC cases (F 1). Moreover, MDR1 expression in HCC tissue sections with nucleic ZHX2 was comparatively lower than that in HCC tissue sections without nucleic ZHX2 (Figure 1). Analysis results of Chi-square test and non-parametric test further confirmed the reverse correlation of nucleic ZHX2 with MDR1 in HCC (Table 1). Both the positive percentage (score of 4–12) and the expression intensity of nuclear ZHX2 (displayed as median ± SD) were significantly lower in MDR1-positive staining samples (score of 4–12) than that in MDR1-negative staining samples (*p* < 0.05). These indicated that reduced nuclear ZHX2 level might be responsible for enhanced MDR1 expression in HCC.

**Table 1:** Immunohistochemical stainning of ZHX2 and MDR1 expression in clinical specimens

		Number of case	ZHX2 expression (Nuclear staining)		
			Positive (4-12)	Negative (0-3)	Median ± SD (range)
MDR1 expression (Membrane staining)	Positive (4-12) Negative (0-3) *p* value	24	5	19	1 ± 2.72
			(20.8%)	(79.2%)	(0-9)
		6	4	2	5 ± 4.42
			(66.7%)	(33.3%)	(1-12)
		0.01 < *p* < 0.05^a^		0.01 < *p* < 0.05^b^

a *p* values were obtained from the *Chi-square test*.

b *p* values were obtained from the *non-parametric test*.

### ZHX2 decreases MDR1 expression and reduces drug efflux from HCC cells

**Figure 2:**
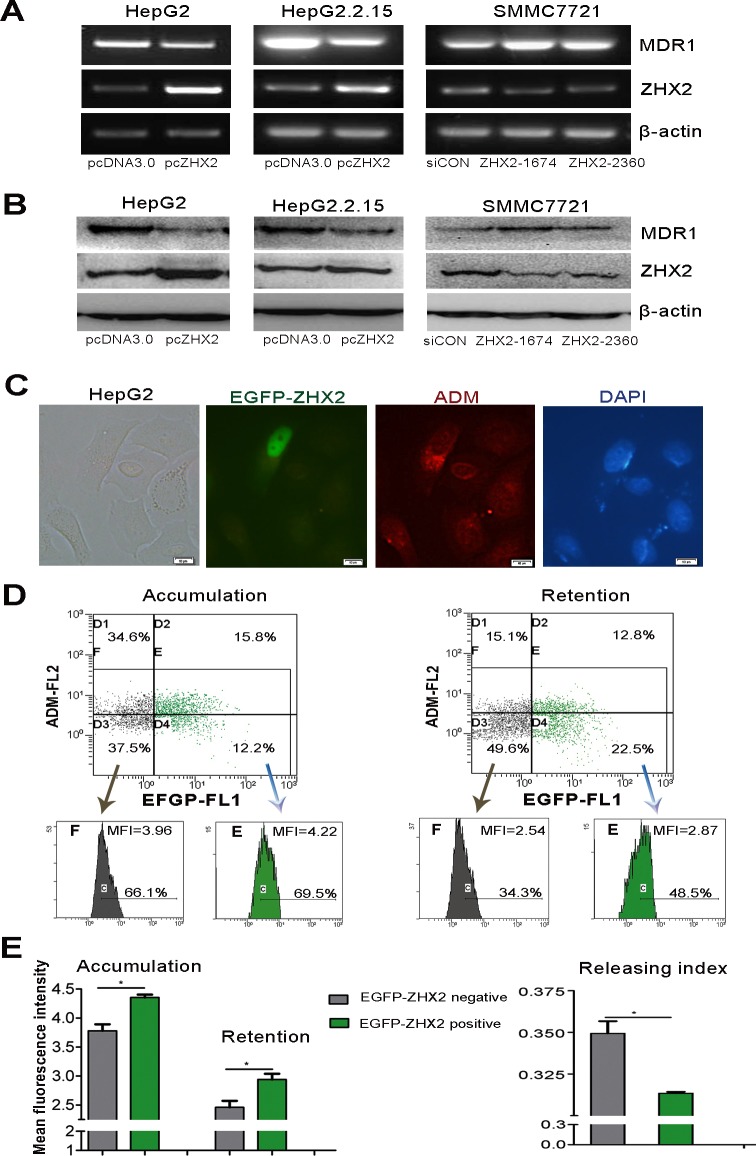
ZHX2 suppresses MDR1 expression and increases ADM retention of HCC cells (A and B) *ZHX2*, *MDR1* and *β-actin* mRNA levels(A) and protein levels (B) were determined by RT-PCR and western blot in HepG2 and HepG2.2.15 cells transfected with pcDNA3.0 or pcZHX2 and in SMMC7721 cells that were transfected with *ZHX2* siRNAs or control siRNA (siCON). (C) ZHX2-EGFP expression and intracellular ADM were determined by fluorescence microscopy of HepG2 cells in pEGFP-ZHX2-transfected cells treated with ADM as described in Methods and Materials. Representative panels are shown. Red, autofluorescence of ADM ; Green, fluorescence of EGFP-ZHX2 ; Blue, DAPI staining. Bars, 10 μm. (D) ADM accumulation (left panels) and retention (right panels) in pEGFP-ZHX2 transfected HepG2 cells were analyzed by flow cytometry. (E) Left panel: ADM accumulation and retention based on data in Figure 2D. Right panel: ADM-releasing index was calculated based on accumulation and retention data from flow cytometry. Data are shown as the mean±SD (n=3); *p < 0.05.

In order to further confirm the negative regulation of *ZHX2* on *MDR1* in HCC, we then did *in vitro* studies. ZHX2 and MDR1 mRNA levels were compared in several liver cancer cell lines. RT-PCR analysis showed an inverse correlation between MDR1 and ZHX2 expression: cells with higher *MDR1* mRNA levels (HepG2 and HepG2.2.15 cells) had lower *ZHX2* mRNA levels whereas those with lower *MDR1* (SMMC7721 cells) had higher *ZHX2* (Figure S1A). Interestingly, ZHX2 expression level correlated with CDDP sensitivity in HCC cells (Figure S1B), indicating that ZHX2 closely correlates with MDR1 expression and chemotherapy sensitivity of HCC cells. To explore further the relationship between these two genes, ZHX2 was overexpressed or knocked down by transient transfection. As shown in Figure 2A, ZHX2 overexpression led to decreased *MDR1* mRNA levels in HepG2 and HepG2.2.15 cells, whereas ZHX2 knockdown with two different siRNAs (ZHX2-1674, ZHX2-2360) resulted in elevated *MDR1* mRNA levels in SMMC7721 cells. This difference was also seen at the protein level as determined by western blot (Figure 2B and Figure S2). These data support the possibility that ZHX2 represses MDR1 expression in HCC cells.

MDR1 is a well-known ATP-dependent drug efflux pump. To evaluate the effect of ZHX2 on regulating the MDR1 transporter activity, HepG2 cells were transfected with pEGFP-ZHX2 and then treated with ADM, which emits a natural red fluorescence. EGFP-ZHX2 expression and ADM autofluorescence intensity were detected by fluorescence microscopy. As shown in Figure 2C, red fluorescence was higher in EGFP-ZHX2 expressing cells than untransfected cells after ADM treatment, indicating greater ADM accumulation in EGFP-ZHX2 transfected cells. Enhanced ADM accumulation in EGFP-ZHX2 expressing cells was further confirmed by flow cytometry. The red MFI in EGFP-positive cells was significantly higher than that in EGFP-negative cells 4 hours after ADM treatment (Figure 2D, left panel). The red MFI in EGFP-positive cells remained higher than EGFP-negative cells 2 hours after ADM withdraw (Figure 2D, right panel), suggesting enhanced ADM retention in EGFP-ZHX2 overexpressing cells. Consistently, EGFP-ZHX2 positive cells exhibited a decreased ADM releasing index compared with EGFP-ZHX2 negative cells (Figure 2E). Taken together, these data suggest that ZHX2 suppresses MDR1 expression and decreases drug efflux, resulting in increased intracellular ADM levels.

### Higher ZHX2 expression increases the cytotoxicity of chemotherapeutic drugs

**Figure 3:**
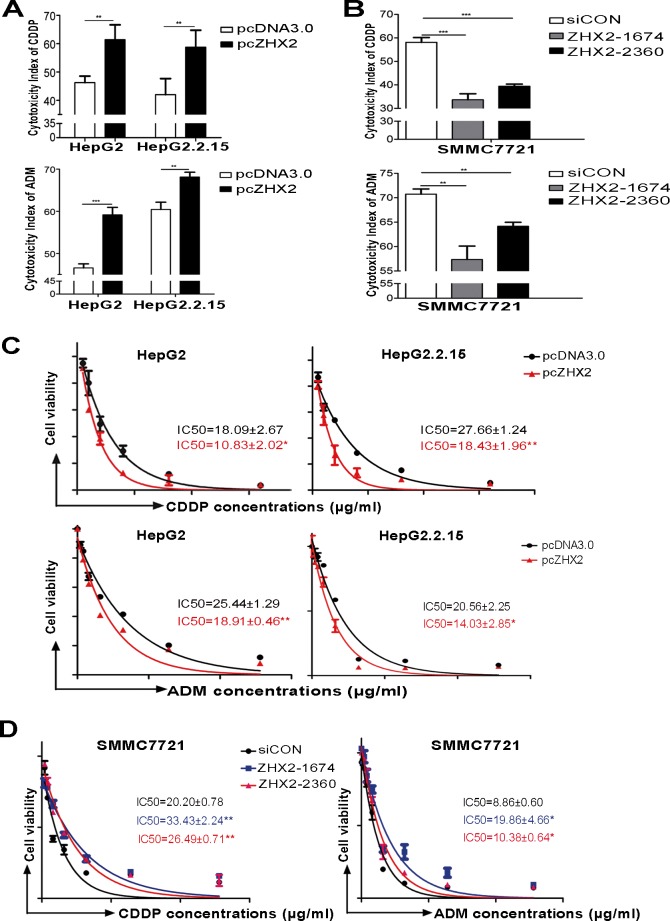
Higher ZHX2 levels increase the sensitivity of HCC cells to the cytotoxic effects of CDDP and ADM HepG2 and HepG2.2.15 cells were transfected with pcDNA3.0 or pcZHX2 (A), whereas SMMC7721 cells were transfected with siCON or *ZHX2*-siRNAs (B). After 24 hours, cells were treated with CDDP (upper panels) or ADM (lower panels) and cultured for another 24 hours. The cytotoxicity was calculated as described in Materials and Methods. Data are shown as the mean±SD (n=4); **p < 0.01, ***p < 0.001. (C and D) IC_50_ of CDDP (upper panels) or ADM (lower panels) in cell lines transfected as described above in A and B. The IC_50_ was calculated as described in Materials and Methods. Data are shown as the mean±SD (n=3); *p < 0.05, **p < 0.01.

The ability of ZHX2 to repress MDR1 led us to consider whether elevated ZHX2 levels would increase drug sensitivity in HCC cells. To test this, the cytotoxicity index of CDDP or ADM was determined in ZHX2-overexpressing cells or ZHX2-knockdown cells. In ZHX2-overexpressing cell lines (HepG2 and HepG2.2.15), the cytotoxicity index increased significantly after treatment with both CDDP and ADM (Figure 3A) compared to pcDNA3.0-transfected cells treated with these drugs. In accordance, knock-down of ZHX2 in SMMC7721 cells decreased the cytotoxicity index of both CDDP and ADM (Figure 3B). This is further supported by IC_50_ assay measured with increasing amounts of CDDP or ADM in different cell populations (Figure 3C and D). These data indicate that increased ZHX2 levels result in increased sensitivity of HCC cells to these chemotherapeutic drugs.

### ZHX2 increases CDDP-induced apoptosis in HepG2 cells

**Figure 4:**
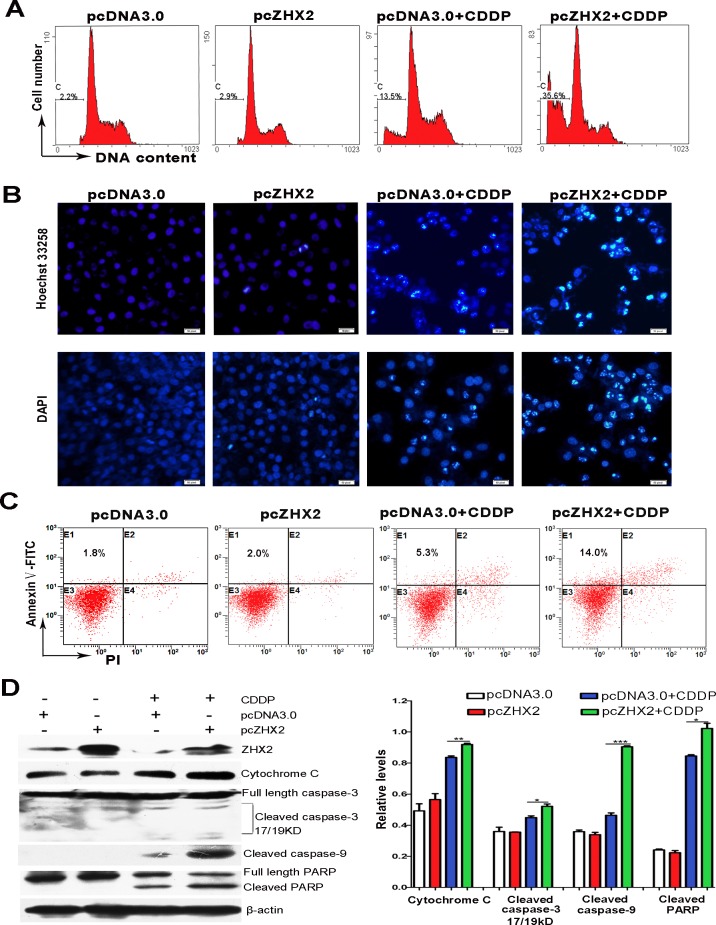
Increased ZHX2 levels in HepG2 cells enhance CDDP-induced apoptosis and activate the caspase pathway HepG2 cells transfected with pcDNA3.0 or pcZHX2 were treated with CDDP for 24 hours. (A) Cells were incubated with PI and analyzed by flow cytometry. The percentage of cells with fragmented DNA (sub-G1 peak) are shown. (B) Staining of cells with Hoechst 33258 (upper images) and DAPI (lower images). Bars, 50 pixel. (C) Flow cytometry after staining with PI and Annexin V to determine the percentage of Annexin V-stained cells. (D) Western blot analysis to determine levels of ZHX2, Cytochrome C, full-length and cleaved caspase 3, cleaved caspase 9, full-length and cleaved PARP, and β-actin. Western blot data from three independent experiments are quantitated in the right panel, with levels if indicated protein shown relative to β-actin. *p < 0.05,**p < 0.01, ***p < 0.001.

Since CDDP acts to increase apoptosis by DNA cross-linking, we next tested whether elevated ZHX2 would enhance CDDP-mediated DNA damage and apoptosis in HepG2 cells as judged by chromatin condensation and nuclear fragmentation [20]. ZHX2 overexpression by itself did not increase the number of apoptotic, sub-G1 cells compared to pcDNA3.0 transfected cells (Figure 4A). As expected, a distinct sub-G1 peak was detected in CDDP-treated HepG2 cells. Interestingly, ZHX2 overexpression significantly increased the sub-G1 population after CDDP treatment, indicating that elevated ZHX2 enhances CDDP-induced apoptosis. This increased apoptosis was further verified by Hoechst 33258 and DAPI staining in HepG2 cells, which showed increased chromatin condensation (Figure 4B) and flow cytometry after staining with Annexin V and propidium iodide (PI, Figure 4C).

To further analyze the effect of ZHX2 on CDDP-induced apoptosis, several apoptosis-related proteins were monitored by western blot. Consistent with earlier studies, CDDP treatment increased levels of cytoplasmic Cytochrome C, cleaved caspase-3, cleaved caspase-9 and cleaved PARP (Figure 4D). Moreover, although ZHX2 overexpression did not alter overall levels of these proteins, pcZHX2 transfection enhanced the CDDP-induced levels of apoptotic products (Figure 4D). This was particularly true for caspase-9, since nearly twice the levels of cleaved caspase-9 were detected in HepG2 cells transfected with ZHX2 and treated with CDDP compared with cells treated with CDDP alone (Figure 4D, right panel). Collectively, these results indicate that ZHX2 overexpression enhanced CDDP cytotoxicity by increasing drug-induced apoptosis in HepG2 cells.

### ZHX2 overexpression enhances CDDP-mediated inhibition of HCC growth in nude mice

**Figure 5:**
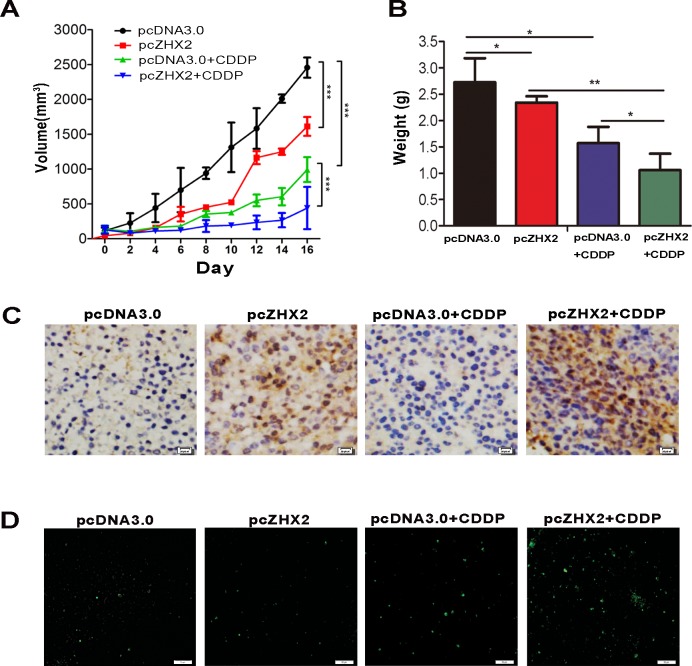
ZHX2 combined with CDDP act cooperatively to inhibit xenograft tumor growth in nude mice Nude mice containing subcutaneous HepG2.2.15 tumors were randomized to receive intra-tumor injections of pcDNA3.0 or pcZHX2, and half of each group were also injected with CDDP. (A) Tumor volume was calculated every other day for the entire 16-day study. Data are shown as the mean±SD ( Two-way ANOVA, ***p < 0.001). (B) Tumor weights in four different cohorts were measured at the end of the experiment. Data are shown as the mean±SD(n≥4); *p <0.05, **p<0.01. (C) Immunohistochemical analysis of ZHX2 expression in tumor samples. Bars, 20 pixel. (D) TUNEL staining as a measure of apoptosis in tumor samples indicate greatest staining in pcZHX2 plus CDDP injected tumors. Bars, 50 μm.

In order to detect whether ZHX2 and CDDP could cooperate to inhibit tumor growth *in vivo*, HepG2.2.15 xenograft tumors were treated with CDDP and/or plasmid DNA (pcDNA3.0 or pcZHX2). As seen in previous studies [16], either CDDP treatment or ZHX2 overexpression alone inhibited xenograft growth significantly (Figure 5A). However, the combination of pcZHX2 and CDDP cooperatively inhibited tumor growth to a greater extent than treatment with pcZHX2 or CDDP alone (Figure 5A). Consistent with the diminished growth curves, tumor weights at time of sacrifice were also significantly reduced in mice with combined treatment compared to mice treated with pcZHX2 or CDDP alone (Figure 5B). Immunohistochemistry staining verified the ZHX2 overexpression in tumors injected with pcZHX2 (Figure 5C). TUNEL staining confirmed that ZHX2 promoted CDDP-induced apoptosis of tumor cells (Figure 5D). These results indicated that ZHX2 overexpression enhances the ability of CDDP to inhibit HepG2.2.15 growth *in vivo*.

### ZHX2-mediated repression on MDR1 promoter activity requires NF-Y

**Figure 6:**
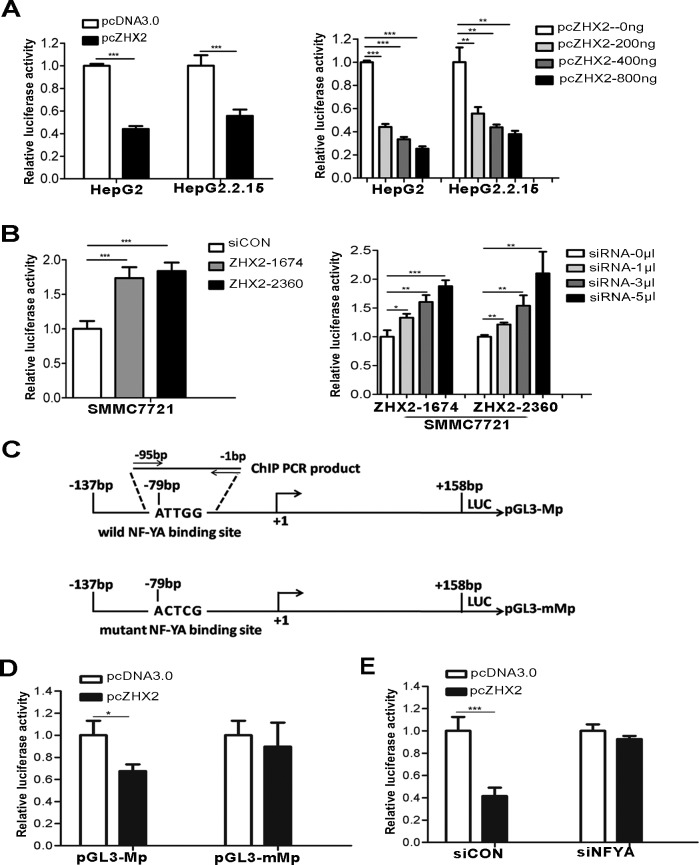
ZHX2 represses *MDR1* promoter activity via NF-YA-dependent interactions (A) HepG2 and HepG2.2.15 cells were co-transfected with pGL3-Mp along with pcDNA3.0 or pcZHX2 (left panel) or with pGL3-Mp and increasing amounts of pcZHX2 (right panel). (B) SMMC7721 cells were transfected with pGL3-Mp and siCON or *ZHX2* siRNAs (left panel) or with pGL3-Mp and increasing amounts of *ZHX2* siRNAs (right panel).(C) Diagram of the wild type and mutant type *MDR1* promoters, showing the location of the *Y box* and the mutation used to generate pGL3-mMp. (D) HepG2 cells were co-transfected with pGL3-Mp or pGL3-mMp along with pcDNA3.0 or pcZHX2.(E) HepG2 cells were co-transfected with pGL3-Mp and pcDNA3.0 or pcZHX2(Left). In addition, cells were also transfected with siCON or *NF-YA* siRNA (siNF-YA, Right). Data are shown as the mean ± SD (n≥3); *p < 0.05, **p < 0.01,***p < 0.001.

Previous studies indicate that ZHX2 functions as a transcriptional repressor [9, 12, 13]. We therefore tested whether ZHX2 represses *MDR1* promoter activity. Results of cotransfection and luciferase assays showed that ZHX2 overexpression significantly decreased *MDR1* promoter activity in HepG2 and HepG2.2.15 cells (Figure 6A). Conversely, ZHX2 knock-down by siRNAs greatly enhanced the *MDR1* promoter activity in SMMC7721 cells (Figure 6B). The decrease and increase in luciferase levels showed a dose-dependent response in cells that were cotransfected with ZHX2 expression vectors or *ZHX2* siRNAs, respectively (Figure 6A and B).

Previous studies identified a NF-YA binding *ATTGG* element (known as *Y box*) in the *MDR1* promoter that is important for full *MDR1* promoter activity [9]. Furthermore, ZHX2 was shown to interact with the NF-YA subunit and inhibit the ability of NF-Y to transactivate target genes [9]. To examine whether ZHX2 regulates *MDR1* promoter activity via the *Y box* in HepG2 cells, ZHX2 cotransfections were performed with wild-type promoter pGL3-Mp or the pGL3-mMp in which the *ATTGG* motif was mutated (Figure 6C). In contrast to pGL3-Mp, the mutant promoter pGL3-mMp was not repressed by ZHX2 (Figure 6D). Furthermore, ZHX2-mediated repression of the *MDR1* promoter was dependent on NF-Y since this repression was no longer evident when NF-YA was knocked down by siRNA (Figure 6E).

**Figure 7:**
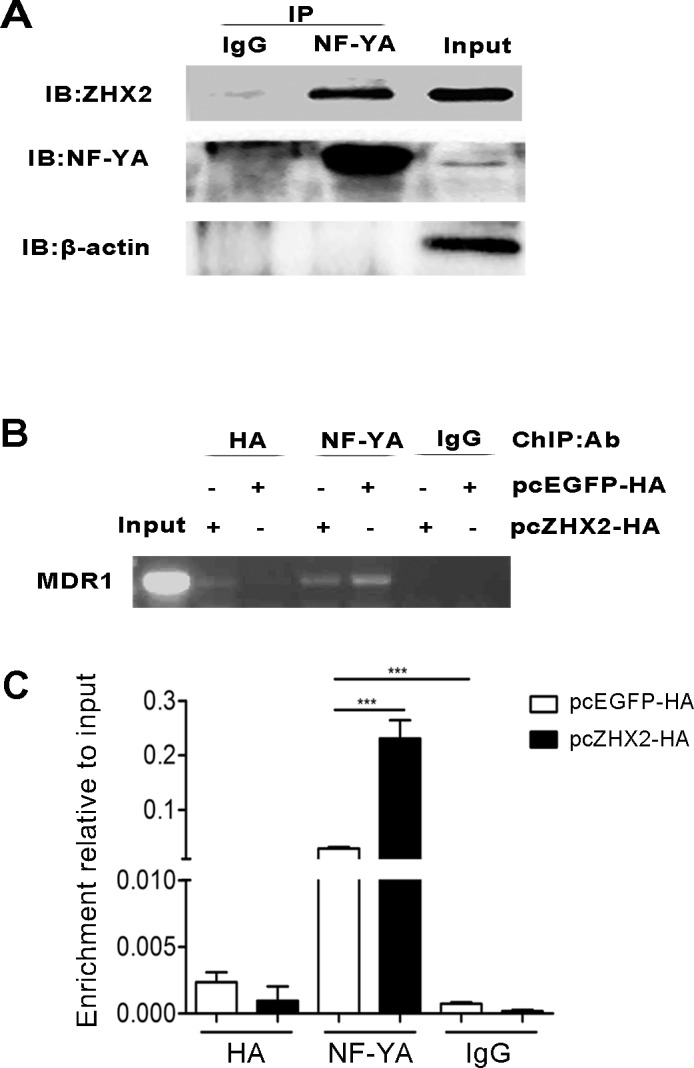
ZHX2 interacts with the *MDR1* promoter via NF-Y (A) Co-IP of NF-YA and ZHX2 after transfection. pcZHX2 plasmid was transfected into HepG2 cells, followed by immunoprecipitations performed as the methods. (B and C) ChIP analysis of DNA from HepG2 cells transfected with pcEGFP-HA or pcZHX2-HA. (B) Conventional PCR amplification of DNA was performed with primers specific to the *MDR1* promoter after immunoprecipitation with anti-HA, anti-NF-YA or IgG (control). (C) DNA enrichment was analyzed at the MDR1 promoter by real-time PCR and the results are presented as fold of enrichment over input. Data are shown as the mean ± SD (n=3); ***p < 0.001.

The transfection data described above led us to consider whether ZHX2 bound directly to the *MDR1* promoter or inhibited NF-Y activity by an indirect mechanism. As previous report in HEK-293, Co-IP demonstrated the interaction of ZHX2 and NF-YA proteins in HepG2 cells (Figure 7A) [9]. To directly test whether ZHX2 bound to the *MDR1* promoter, ChIP were performed using HepG2 cells transfected with pcZHX2-HA or pcEGFP-HA and oligonucleotides specific for the *MDR1* promoter (Figure 6C). As shown in Figure 7B, a specific PCR amplification was detected in the anti-HA immunoprecipitation from HepG2 cells transfected with pcZHX2-HA but not in pcEGFP-HA, indicating that ZHX2 bound to the *MDR1* promoter. Interestingly, a relative weak PCR amplification was detected in the anti-NF-YA immunoprecipitation from HepG2 cells transfected with pcZHX2-HA than that in pcEGFP-HA (Figure 7 B and C), suggested that the presence of transfected ZHX2 could influence the NF-YA binding to the MDR1 promoter. Taken together, these data suggest that ZHX2 interacts directly with NF-Y on the *MDR1* promoter and that this interaction inhibits NF-Y-mediated activation of *MDR1* transcription.

## DISCUSSION

It is well established that MDR1/Pgp is a drug efflux pump responsible for the transport of a variety of antineoplastic drugs from the cells. Elevated MDR1 is strongly implicated in MDR and decreases the efficacy of cancer chemotherapy [3, 21]. Inhibition of MDR1/Pgp has been proposed as a powerful way to overcome efflux-mediated chemotherapy resistance in cancer cells [3, 4]. Here, we identified the ubiquitous transcription factor ZHX2 as a negative regulator of *MDR1* transcription. Increased ZHX2 levels led to reduced drug efflux and enhanced the cytotoxicity of anti-cancer drugs in HCC cell lines. Elevated ZHX2 levels significantly enhanced CDDP mediated suppression of liver tumor growth *in vivo*. These data are consistent with previous clinical studies demonstrating an association between ZHX2 expression and enhanced survival of patients with multiple myeloma after chemotherapy treatment [17, 22].

ZHX2 was originally identified based on its interactions with ZHX1 and NF-Y [9]. Subsequently, ZHX2 was found to repress the expression of genes that are frequently upregulated in HCC, including AFP, GPC3 and H19 [11, 12]. We recently identified cyclin A and cyclin E as ZHX2 targets [16]. Here, we added MDR1 as a new target of ZHX2. Based on our results, we propose that ZHX2 represses MDR1 transcription by interacting with NF-YA that is bound to the *CCAAT* box in the *MDR1* core promoter region. Previous studies demonstrated that NF-Y was an important regulator of MDR1 expression [6]. Our transient transfections confirmed the importance of the *CCAAT* box (Figure 6D) and the requirement of NF-Y (Figure 6E) for ZHX2-mediated repression. The co-immunoprecipitation assays confirmed previous reports showing interactions between ZHX2 and NF-YA (Figure 7A) [9]. This interaction between ZHX2 and NF-YA on the *MDR1* promoter was further verified by ChIP analysis (Figure 7 B and C). Previous studies identified NF-Y as a major component of *MDR1* transcriptional complex, termed the MDR1 “enhancesome”, which is responsible for regulation of MDR1 transcription by a variety of stimuli, such as UV irradiation, HDAC (histone deacetylases) inhibitors and certain chemotherapeutics [23-25]. Moreover, NF-Y is required for recruitment of the histone acetyltransferase P/CAF or histone methyltransferase specific for H3K4 MLL1(mixed lineage leukemia 1) to the *MDR1* promoter, resulting in the transcriptional activation that is likely mediated by further chromatin remodeling [24, 26]. Whether ZHX2 disturb the binding of the enhancesome and the recruitment of chromatin-modifying factors to the *MDR1* promoter will require further studies.

Our data presented here demonstrate that ZHX2 inhibits MDR1 expression, which promotes the intracellular accumulation of chemotherapeutic drugs and increases their cytotoxic effects in HCC. This model is supported by ADM accumulation in HepG2 cell (Figure 2C). The combined effect of ZHX2 and CDDP in the inhibition of tumor growth *in vivo* further supports the hypothesis. CDDP is generally believed to kill cancer cells by binding to DNA and interfering with cellular DNA repair mechanism, which eventually cause apoptosis [27, 28]. As expected, we detected CDDP induced apoptosis in cultured HCC cells and HepG2.2.15 cells grown in nude mice. Although ZHX2 transfer alone did not cause obvious apoptosis, ZHX2 overexpression significantly increased CDDP-induced cell apoptosis both *in vitro* and *in vivo* (Figure 4 and 5D). In addition, western blots showed that combination of ZHX2 overexpression and CDDP led to increased release of cytochrome c and enhanced cleavaged caspase-3, caspase-9 and PARP (Figure 4D). This is consistent with the previous report which demonstrated the CDDP induced mitochondria-driven apoptosis in tumor cells [29]. Although chemotherapeutic agents exert their effects on tumor cells through various mechanisms, apoptosis is likely the final pathway shared by most chemotherapeutic agents [30, 31]. Nagel et al. found that ZHX2 activated STAT1 signaling, which could contribute to apoptosis [15]. This raises the potential that ZHX2 promotes chemotherapy effects by regulating apoptosis related genes and might lead to efflux-independent chemotherapy resistance in HCC cells.

We recently reported that ZHX2 inhibits Cyclin A and Cyclin E expression, leading to HCC cell cycle arrest [16]. This indicates that ZHX2 functions as a tumor suppressor in HCC. Our data provided here indicates that ZHX2 represses MDR1 expression and therefore enhances the effect of chemotherapy via increased intracellular drug concentration. This is therefore a second mechanism by which ZHX2 influence HCC cell growth. Further investigation using clinical specimens will be required to determine whether abnormal ZHX2 expression in liver tumors may be used as a potential biomarker for predicting chemotherapeutic drug resistance. Our data also suggest that increasing ZHX2 levels may decrease the growth of HCC cells and increase their sensitivity to chemotherapeutic agents. Identifying additional ZHX2 targets may also elucidate other HCC therapy targets. In conclusion, the results provide a basis for further clinical research in combining ZHX2 and chemotherapeutic agents to treat liver cancer.

## MATERIALS AND METHODS

### Cell lines, plasmids and siRNAs

The human HCC cell lines SMMC7721 were cultured in RPMI 1640. Human hepatoma cell lines HepG2 were cultured in minimum essential medium (MEM) with 1mmol/L sodium pyruvate. HepG2.2.15 cells were cultured in MEM with 380 μg/ml G418 (GIBCO). Human embryonic kidney 293 (HEK293) cells were cultured in Dulbecco's modified Eagle's medium. All the cells were purchased from Shanghai Institute of Cell Biology (Chinese Academy of Sciences, Shanghai, China), and all the media were supplemented with 10% fetal bovine serum (FBS, GIBCO).

ZHX2 expression vectors pcZHX2 and pEGFP-ZHX2 and the siRNAs against ZHX2 (ZHX2-1674, ZHX2-2360) were described previously [16]. pcEGFP-HA was constructed by cloning the EGFP-HA to the pcDNA3.0 plasmid. The promoter regions of human MDR1(−137 to +158, the transcription initiation site designated as +1) was cloned into the promoterless pGL3-basic vector (Promega) to prepare luciferase reporter plasmid (pGL3-Mp). Reporter plasmid containing mutant MDR1 promoter (pGL3-mMp) was constructed by mutating the wild-type *Y-box* (*ATTGG* to *ACTCG*). The siRNA against NF-YA was purchased (sc-2997, Santa Cruz, CA, USA).

### RT-PCR and Western blot

Total RNA extracted with TRIzol reagent (Invitrogen) were reverse transcribed into cDNAs using a Thermo Scientific RevertAid First Strand cDNA Synthesis Kit. Conventional PCR was carried out in a BioRad Thermal Cycler with specific primers (Table S1).

Cytoplasmic extracts and whole cell extracts were prepared as described previously [16]. 40 μg of protein separated by SDS-PAGE were bloted with following antibodies: anti-ZHX2 (ab56886, Abcam, MA, USA), anti-β-actin (sc-1616-R, Santa Cruz, CA, USA), anti-MDR1(sc-55510, Santa Cruz, CA, USA) and anti-cytochrome c (4280), anti-caspase-3 (9668), anti-caspase-9 (9508), anti-PARP (9532) from Cell Signal Technology, MA, USA.

### Cytotoxicity assay

To evaluate the effect of ZHX2 on the toxicity of chemotherapeutic drugs, ZHX2-overexpressing cells or ZHX2-knockdown cells were treated with Cisplatin (CDDP, 20μg /ml; QiLu Pharmaceutical, Jinan, China) or Adriamycin (ADM, 20 μg/ml; Pfizer Pharm, NY, USA) for 24 hours. Cell viability was assayed using the Cell Counting Kit-8 (CCK-8, Dojindo, Shanghai, China). The cytotoxicity index was calculated as (1 - OD_450_ of drug-treated cells / OD_450_ of untreated cells) × 100%. The IC_50_ values of CDDP and ADM were calculated using GraphPad Prism 5 (Version 5.01, GraphPad Software, San Diego, CA) [32].

### Cell cycle and apoptosis analysis

Cells transfected with pcZHX2 or pcDNA3.0 were treated with CDDP (20μg/ml) for 24 hours. Apoptotic cells were estimated either by Hoechst 33258 (1 μg/ml, Promega) or DAPI (1 μg/ml, Promega) staining or flow cytometry with the Apoptosis detection kit (BU-AP0103, Biouniquer Technology Co, Ltd, Nanjing, China). The cell cycle were anlayzed by flow cytometry after propidium iodide (PI, Sigma, USA) staining. Flow cytometery were performed with Beckman Coulter Flow Cytometer (Miami, USA).

### Clinical samples and immunohistochemical staining

To estimate the correlationship of ZHX2 and MDR1 in HCC, ZHX2 and MDR1 immunohistochemical staining was performed in HCC tissues from 30 HCC patients (Table S2) who underwent surgery between 30 October 2013 and 29 August 2014 at Qilu Hospital and Shandong Provincial Hospital affiliated to Shandong University (Shandong, China). None of the patients was positive for HCV or HIV. Informed consent was obtained from all patients before the study was initiated with approval of the Shandong University Medical Ethics Committee in accordance with the Declaration of Helsinki.

Immunohistochemical staining using anti-ZHX2 (ab56886, Abcam, MA, USA) and anti-MDR1 (sc-55510, Santa Cruz, CA, USA) antibody was performed and analyzed as described previously [16]. Eight fields of ~1000 cells from each HCC sections were counted independently by three pathologists. Nuclear ZHX2 staining and membrane MDR1 staining were reported separately according to the German semi-quantitative scoring system [33, 34]. Briefly, each sample was scored according to staining intensity (no staining = 0; weak staining = 1; moderate staining = 2; strong staining = 3) and the number of stained cells (0% = 0; 1-25% = 1; 26-50% = 2; 51-75% = 3; 76-100% = 4). Final immunoreactive scores were determined by multiplying the staining intensity by the number of stained cells, with minimum and maximum scores of 0 and 12, respectively [35].

### *In vivo* xenograft tumor studies

Male Balb/c nude mice (4~6 weeks of age) were purchased from the Animal Research Committee of Institute of Biology and Cell Biology (Shanghai, China) and housed under specific pathogen-free conditions according to protocols approved by the Shandong University Animal Care Committee. *In vivo* studies were performed as described previously [16]. Briefly, HepG2.2.15 cells xenografts were prepared and treated by intratumoral injection of pcZHX2 (20 μg) or pcDNA3.0 (20 μg) together with CDDP (80 μg) at 3 days interval for four times. The tumor volume and tumor weight were estimated. Animal experiments(6 mice per group) were repeated at least twice.

### TUNEL assays

Detection of apoptosis in mouse tumor tissues was carried out by terminal deoxyribonucleotidyl transferase-mediated dUTP nicked labeling (TUNEL) analysis, and was performed according to the manufacturer's protocol (In Situ Cell Death Detection Kit, Fluorescein Roche, Cat. No. 11684795910).

### Intracelluar drug accumulation and retention assay

The natural red fluorescence of the chemotherapeutic drug ADM was used to determine drug accumulation and retention[36]. Briefly, HepG2 cells transfected with pEGFP-ZHX2 were cultured in medium with 40μg/ml ADM for 4 hours at 37^o^C. Drug accumulation in the cells was estimated either by fluorescence microscope or flow cytometry. For flow cytometry, cells were gated as EGFP-positive and EGFP-negative, then red MFI of the gated cells was used to determine drug accumulation. To measure the drug retention, ADM treated cells were cultured with fresh, drug-free medium for another 2 hours allowing drug efflux from cells. The ADM releasing index of HepG2 cells was calculated as following: releasing index = (accumulation MFI-retention MFI)/accumulation MFI.

### Co-immunoprecipitation(Co-IP)

Cell lysates from HepG2 cells transfected with pcZHX2 were precipitated with control IgG (sc-2027, Santa Cruz, CA, USA) or NF-YA antibody (ab6558; Abcam, Hong Kong). After incubation with protein A/G Plus-Agarose(sc-2003, Santa Cruz, CA, USA), the precipitates were immunobloted with anti-HA antibody (ab9110; Abcam).

### Chromatin immunoprecipitation(ChIP) assays

ChIP assays was performed with HepG2 cells transfected with pcZHX2-HA or pcEGFP-HA as reported [16]. Briefly, fixed cells were sonicated to shear DNA to 200~1000 bp and immunoprecipitated using anti-HA antibody (ab9110; Abcam), anti-NF-YA antibody (ab6558; Abcam) or rabbit IgG (sc-2027, Santa Cruz, CA, USA). As controls, 1/20th of the starting chromatin (Input) was used. Analysis was performed using specific primers for the NF-YA binding region of *MDR1* promoter.

### Real-time quantitative PCR

Real-time quantitative PCR was carried out in BioRad C1000^TM^ Thermal Cycler CFX96^TM^ Real-Time System using SuperReal PreMix Plus (SYBR Green, TIANGEN BIOTECH, BEIJING). The results are presented as fold of enrichment over input. The used primers were shown in Table S1.

### Luciferase reporter assays

HCC cell lines were transfected with reporter plasmids (0.2μg) and pcZHX2(0.4μg) or *ZHX2* siRNAs using Lipofectamine^TM^ 2000(Invitrogen). To further explore the role of NF-YA in ZHX2 mediated control of the *MDR1* promoter, HepG2 cells were co-transfected with reporter plasmids, *NF-YA* siRNA and pcZHX2. Luciferase reporter assay was performed using Dual-Luciferase Reporter Assay System (Promega).

### Statistics

GraphPad Prism (GraphPad Software, San Diego, CA) was used for data analysis. Data values were presented as the means ± SD. The statistical correlation between the MDR1 staining levels and the ZHX2 staining levels in tissue sections was analyzed by the Chi-square test. Two-way ANOVA followed by Bonferroni post-tests was applied to determine significant differences between different treatments in xenograft tumor studies. The Student t-test was applied to determine significant differences between groups. In these analyses, *p* <0.05 were considered to statistically significant.

## SUPPLEMENTARY MATERIAL AND FIGURES



## Abbreviations

ZHX2: Zinc-fingers and homeoboxes 2
HCC: hepatocellular carcinoma
MDR: multidrug resistance
CDDP: cis-diamminedichloroplatinum
ADM: Adriamycin
