# Supplementary material for: ZHX2 enhances the cytotoxicity of chemotherapeutic drugs in liver tumor cells by repressing MDR1 via interfering with NF-YA
Source: Oncotarget. 2014 Nov 26;6(2):1049–63. doi: 10.18632/oncotarget.2832 (PMC4359216; doi:10.18632/oncotarget.2832)
Supplement: Supplementary file 1 [file oncotarget-06-1049-s001.pdf]

ZHX2 enhances the cytotoxicity of chemotherapeutic drugs in liver tumor cells by repressing MDR1 via interfering with NF-YA

Supplementary Material

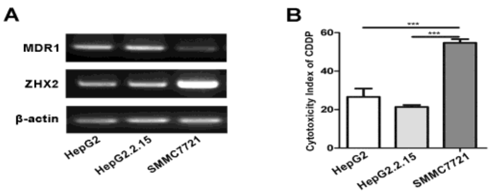

Figure S1: ZHX2 expression is closely related to the expression of MDR1 and cytotoxicity of chemotherapeutics. (A) Total RNA was extracted from HepG2, HepG2.2.15 and SMMC7721 cell lines. RT-PCR was used to analyze ZHX2 and MDR1 mRNA levels. The β-actin mRNA levels were used as a control. (B) These cell lines were treated with CDDP and cultured for 24 hours. At this time, the number of viable cells was determined to calculate the cytotoxicity as described in Materials and Methods. Data are shown as the mean±SD (n=4); ***p < 0.001.

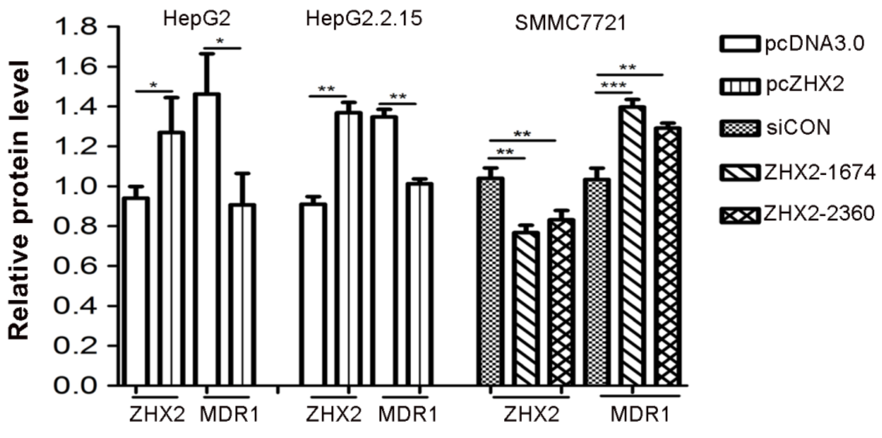

Figure S2: ZHX2 suppresses MDR1 expression in HCC lines. HepG2 and HepG2.2.15 cells were transfected with pcDNA3.0 or pcZHX2, while SMMC7721 cells were transfected with

ZHX2 siRNAs or control siRNA (siCON). ZHX2, MDR1 and β-actin protein levels were determined by western blot. ZHX2/ β-actin and MDR1/ β-actin protein ratios were quantified by Quantity One® Software (Bio Rad) and analyzed by unpaired t test. Data are shown as the mean±SD (n=3); **p* < 0.05, ***p* < 0.01, ****p* < 0.001.

Table S1: Primer sequences for RT-PCR, ChIP-PCR and vector construction.

**Name of primer**	**Forward sequence**	**Reverse sequence**
Human β-actin RT-PCR	AGTTGCGTTACACCCTTTC	CCTTCACCGTTCCAGTTT
Human ZHX2 RT-PCR	CCCCCAATGGTGCTCTGT	TTGCTTTCCTTGCTACGG
Human MDR1 RT-PCR	GGTTTATAGTAGGATTTACACGTGGTTG	AAGATAGTATCTTTGCCCAGACAGC
pGL3-Mp	CGCAGTTTCTCGAGGAATCAGCATTCAGTCAATCC	GGCAAGCTTAGTAGCTCCCAGCTTTGCGTGCCCCTAC
pGL3-mMp	CTGTGGTGAGGCTGAcTcGCTGGGCAGGAACAG	CTGTTCCTGCCCAGCgAgTCAGCCTCACCACAG
MDR1 ChIP	ATCTGTGGTGAGGCTGATTGG	GAGCTCAGGCTTCCTGTGGC
pcEGFP-HA	GGGGTACCATGGTGAGCAAGGGCGAGGAGC	CGGAATTCCCTAAGCGTAGTCTGGTACGTCGTAAGGGTA-<br>-CTTGTACAGCTCGTCCATGC

Table S2: Clinical characteristics of HCC samples.

Sample characteristics	Number of cases
**Number of patients**	30
**Age (yr; median, range)**	50, 34-79
**Gender (male/female)**	23/7
**HbsAg (negative/positive)**	6/24
**Cirrhosis (absent/present)**	5/25
**ALT (U/liter; ≤42/>42)**	12/18
**AFP (ng/ml; ≤25/>25)**	6/24
**Tumor size (cm; ≤5/>5)**	13/17
**Differentiation grade (I-II/II-IV)**	16/14
**TNM stage (I+II/III+IV)**	13/17

HbsAg, hepatitis B surface antigen; ALT, alanine aminotransferase; AFP, α-fetoprotein; TNM, tumor node metastasis.
